# The impact of life events on health-related quality of life in rural older adults: the moderating role of social support

**DOI:** 10.3389/fpubh.2025.1587104

**Published:** 2025-06-06

**Authors:** Lijun Liu, Jiaman Li, Yiwen Tang, Cheng Chen, Chan Yu, Xiaofeng Li, Li Peng, Daikun Zheng

**Affiliations:** ^1^School of Public Health and Management, Chongqing Three Gorges Medical College, Chongqing, China; ^2^Department of Basic Education, Chongqing Energy Industry Technician College, Chongqing, China; ^3^Medical Examination Center, Chongqing General Hospital, Chongqing, China; ^4^Department of Medical Administration, Dianjiang County General Hospital, Chongqing, China; ^5^School of Marxism, Chongqing Three Gorges Medical College, Chongqing, China

**Keywords:** health-related quality of life, life events, social support, moderating effect, older adult

## Abstract

**Background:**

Global aging is one of the most significant social trends of the 21st century. Health-Related Quality of Life (HRQoL) is an important indicator not only for evaluating the effectiveness of medical interventions, public health policies, and disease management, but also for enhancing the health of the older adult. This study aimed to explore the mechanisms through which life events and social support affect the HRQoL of rural older adults, with the aim of promoting healthier aging in this population.

**Methods:**

A stratified random sampling method was used to select rural older adult individuals from southeastern Henan Province, China. Data were collected using the Life Events Scale, the Social Support Scale (SSS), and the SF-8 Health Survey. T test and the analysis of variance were employed to compare characteristics differences in HRQoL. Pearson’s correlation analysis was used to assess the relationships between life events, social support, and HRQoL. And a regression test was used for Moderating Effect analysis.

**Results:**

The participants in this questionnaire survey showed that Univariate analysis revealed statistically significant associations between age, education, chronic disease in HRQoL among the rural older adult. *Pearson* correlation analysis revealed significant negative correlations not only between life events and social support, but also between life events and HRQoL, while a significant positive correlation was found between social support and HRQoL. Regression analysis for moderating effects showed that life events negatively predicted HRQoL, while social support positively predicted HRQoL. The interaction term of the product life events and social support was also significant.

**Conclusion:**

Our results confirm that life events and social support were significant predictors of the HRQOL among rural older adults, with social support acting as a moderating variable, and provide empirical evidence that enhancing social support systems and reducing negative life events are crucial for improving the health-related quality of life in rural older adult populations and achieving healthy aging.

## Introduction

According to the National Bureau of Statistics of China, by the end of 2023, the number of people aged 60 or above in China had reached 280 million, accounting for 19.8 percent of the total population. China’s aging shows the characteristics of large scale, fast speed, and the phenomenon of “getting old before getting rich.” Rural regions confront more pronounced aging trends, characterized by relatively underdeveloped older adult care infrastructures, which present significant challenges to the health and quality of life for the older adult population in these areas. To proactively address the challenges of population aging and promote healthy aging, the Chinese government has recently issued a series of policy documents, thereby establishing a comprehensive policy framework. Policy documents, including the National Medium- and Long-Term Plan for Actively Responding to Population Aging and the 14th Five-Year Plan for the Development of Aging Services and older adult Care Systems, have delineated strategic objectives, key initiatives, and measures from a high-level design perspective. These documents emphasize critical areas such as older adult care and health services, thereby facilitating the effective implementation of relevant policies.

Health-related quality of life (HRQoL) encompasses an individual’s comprehensive experience and perception of health across physical, psychological, social functioning, and mental states. It extends beyond the treatment and prevention of diseases to emphasize overall satisfaction and functional status in both healthy and diseased states. HRQoL is a critical metric for evaluating population health, particularly in aging societies, where it serves as a key indicator of the overall well-being of older adult populations. Given the limitations of medical resources, economic constraints, and inadequate social support, rural older adult individuals often encounter significant challenges in maintaining their HRQoL. In 2022, Chile has experienced rapid population aging, but the social security system has failed to keep up, resulting in greater social inequality for the older adult, and self-rated health was also significantly associated with quality of life (1). Investigating the health-related quality of life (HRQoL) of rural older adult populations and identifying its influencing factors can facilitate the development of targeted interventions aimed at improving health outcomes, promoting health equity, and supporting active aging. Additionally, HRQoL encompasses an individual’s subjective experience of physical, psychological, social functioning, and overall well-being. This research holds substantial practical value, particularly in light of China’s rapidly aging population.

Social support is an essential concept in psychology, sociology, and public health, denoting the material, emotional, and informational assistance that individuals receive from their social networks. The concept includes emotional, instrumental, informational, and appraisal support, all of which play a critical role in stress management, mental health enhancement, and quality of life improvement. According to Lu et al. (2) research, it divides social support into formal and informal, and no matter whether formal or informal social support, both can improve the HRQoL of rural older adults in China, with the quality of support being more important than the quantity (2). In 2020, León et al. highlighted that the availability of social resources can reduce depression, improve explicit memory, and enhance the perception of quality of life (3). In 2021, the study emphasized that social support has a positive impact on the overall health perception of the older adult, particularly through enhancing life (4), and is a significant factor influencing the physical and mental health of older adult migrants, positively correlating with HRQoL (5). In 2024, the research among older adults in Nigerians found a significant correlation between social support and quality of life, with support from family and significant others having a substantial impact on mental health and quality of life (6). It can be seen that social support plays an important role in the quality of life among the older adult, especially in rural areas.

Life events refer to specific incidents or situations experienced by individuals in their daily lives, encompassing various aspects such as work, education, family, and social interactions. Life events are an essential part of daily life and have profound effects on an individual’s psychology and behavior. These events can be positive, such as promotions or marriages, or negative, such as job loss or illness. Positive events generally elicit positive emotional responses and beneficial outcomes in individuals, whereas negative events tend to impose pressure, anxiety, pain, and other adverse emotional states along with detrimental effects. Wang et al. (7) found that physical activity levels are positively correlated with individual mental and physical health before and after traumatic events (7). In 2020, it found that traumatic life events have a significant direct impact on the HRQoL of 2,987 HIV patients (8). And traumatic life events increase psychological distress and suicidal tendencies (9). Lifelong exposure to traumatic events is negatively correlated with the physical and mental health-related quality of life of the older adult (10). Thus, life events, especially traumatic ones, have a significant negative impact on the quality of life of the older adult. Life events are an inevitable part of life, and occurrence prompts people to constantly adjust their living and mental states to adapt to new circumstances and changes. Understanding and effectively coping with these events can help improve quality of life.

Health-related quality of life is a multidimensional concept that includes physical, psychological, and social adaptation, reflecting an individual’s overall health status and daily functioning. In daily life, various life events can affect an individual’s health, such as biological, physical, or environmental stressors, financial difficulties, and interpersonal challenges. Social support refers to the material, emotional, and informational assistance provided by family, communities, social organizations, and the government. When individuals face stressful or negative life events, receiving more support from social systems can improve their HRQoL. Through a cross-sectional survey among 449 Hong Kong new immigrants from Mainland China, perceived social support and optimism were two important factors to improve the quality of life of immigrants, and in order to improve the quality of life of new immigrants, it should be enhanced their perceived social support (11). According to a survey in 2024, stressful life events may trigger tinnitus, and then be dangerous to health (12). With the moderating effect of family support and social activities, the mean difference in depression caused by stressful life events in the older adult was reduced, then social support, including family support and social activities, buffers the harmful effects of depression caused by stressful life events (13). Previous studies have not shown how life events affect health-related quality of life in older adults. In 2024, a study suggested that social support, particularly from family, plays a crucial role in moderating the relationship between exposure to traumatic events and mental health-related quality of life (10). Therefore, for older adult individuals experiencing physical and psychological decline, receiving support from family and social systems when facing various life events can improve their health. This study focuses on how to improve the quality of life in later life, especially in old age, in the face of some life events such as physical decline, social role change, and increased loneliness.

Presently, a significant proportion of older adult individuals in China reside in rural regions. As they age and confront chronic conditions stemming from earlier life stages, their capacity for labor diminishes, resulting in decreased income. In these circumstances, social support systems assume critical importance. When rural older adults receive increased social support, the material and spiritual support for the older adult will increase, and the older adult will be less affected when facing negative life events, which and the multi-dimensional health indicators of the older adult will also be developed.

This study focused on rural older adults in China, aiming to uncover how life events and social support impact their health—related quality of life (HRQoL). It deeply explored the influence paths of life events on HRQoL in rural older adults. By examining life events in family, health, and social interaction aspects, the research determined their effects on quality of life among rural older adults, as well as the impact degree and direction. Moreover, the role of social support in the relationship between life events and HRQoL in rural older adults would be investigated. Whether social support could moderate the impact of life events on the quality of life would be examined. When a moderating effect existed, the specific ways would be further clarified. The research aimed to understand these relationships better, providing valuable insights for improving well-being among rural older adults and promoting healthy aging. The health-related quality of life of rural older adults is not only connected to rural revitalization and new rural construction but also to the long-term development and realization of the “Healthy China 2030” initiative.

## Materials and methods

### Research objectives

In this study, the primary objective was to examine the impact of life events on the health-related quality of life (HRQoL) of rural older adult individuals in China, with particular attention to the moderating role of social support. The study sought to elucidate how life events and social support interact to influence HRQoL, thereby uncovering potential strategies to enhance the well-being of this vulnerable population. The secondary objective was to identify demographic factors (e.g., age, education level, presence of chronic diseases) associated with HRQoL in this population and to evaluate the moderating effect of social support on the relationship between life events and HRQoL.

### Research hypotheses

**Figure 1:**
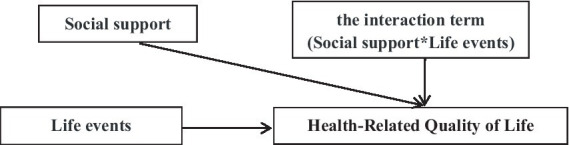
A model of the influence of life events and social support on health-related quality of life in the older adult.

This study concentrates on rural older adults to investigate the factors that influence their HRQoL and proposes the following hypothesis. Null hypothesis Ho: There is no significant association between life events and HRQoL among rural older adults, and social support does not moderate this relationship. Research hypothesis Ha: Primary Hypothesis is that life events and social support are significantly associated with lower HRQoL in rural older adults. The Moderation Hypothesis is that social support moderates the relationship between life events and HRQoL. The specific model is shown in Figure 1.

### Study setting and duration

In this study, the stratified random sampling method was used to randomly select three townships in Wancheng District of Nanyang, Henan Province. Villages within each township were arranged in alphabetical order, and every third village was selected. Finally, older adult individuals aged 60 and above with clear communication abilities were selected, excluding those with severe cognitive impairments (assessed via a brief cognitive screening). This approach balanced geographic and demographic diversity.

Before the formal investigation, the investigators conducted questionnaire-filling training for prospective participants. After the questionnaires were collected, the investigators checked the completeness, internal consistency, and rationality of the questionnaire. Before statistical analyses, we conducted strict quality control on the questionnaire to ensure its effectiveness. Data were removed if they met one of the following criteria: incomplete data; anomalous data (e.g., selection of all the same options, and the illogical or disorderly selection of options).

The study was conducted between January 26, 2024, and February 22, 2024. A total of 750 questionnaires were distributed, with 740 returned, yielding a response rate of 98.67%, and 729 were valid, resulting in an effective rate of 98.51%. Before beginning the study, ethical approval was obtained from Chongqing Three Gorges Medical College of Science & Technology. All subjects provided informed consent before participating in the study, and participant confidentiality was strictly maintained throughout the study duration.

### Questionnaire

The questionnaire used in this study was revised after an in-depth review of the relevant literature and discussion by experts, including professors and chief physicians who have long been engaged in public health management, and health education. The questionnaire was divided into four parts: basic demographic data, social support, life events, and health-related quality of life.

Life events were measured using a scale suitable for the older adult that was compiled and revised by Chinese scholar Xiao (24), and it has good reliability and validity, including events in family life, life events in social interactions, and personal health and so on. Life events are scored that the degree of impact of the event, the time of impact, and the frequency of the event, are multiplied. Cronbach’s α of the questionnaire was 0.922.

Social support was measured using the Perceived Social Support Scale (PSSS) developed in 1986 by Zimet (14). The scale has good reliability and validity, consists of 12 items that rated on a seven-point Likert scale from “strongly disagree” to “strongly agree.” The total score represents the level of social support received. Cronbach’s α of the questionnaire was 0.924.

Health-related quality of life was measured using the widely used SF-8 Health Survey (15). The SF-8 is a concise scale with fewer items and a simple structure, making it easier for older adult individuals to understand. And it presented good reliability and validity. Scores were calculated as (raw score − minimum possible score)/(maximum possible score − minimum possible score) * 100. Higher scores indicate better HRQoL. The Cronbach’s α of the questionnaire was 0.916.

### Statistical analyses

SPSS 25.0 was used to analyze the data. Categorical data are presented as frequencies and percentages, and quantitative data are presented as mean values and standard deviations (for normally distributed data). One-way analysis of variance (ANOVA) and the non-parametric test were used to compare HRQoL among different categories of participants. Pearson’s correlation analysis was used for multi-factor analyses to assess the correlations between social support, life events, and health-related quality of life, and for the generalized linear analysis. A *p*-value of <0.05 was considered statistically significant.

## Results

### Basic characteristics of the scores in HRQoL

Table 1 shows the basic characteristics and HRQoL scores of the participants surveyed. Independent sample t-tests revealed no significant differences in HRQoL based on gender (*t* = 0.54, *p* > 0.05) or participation in recreational activities (*t* = −1.02, *p* > 0.05). However, significant differences were found based on the presence of chronic diseases (*t* = 21.70, *p* < 0.05), with older adult individuals without chronic diseases reporting higher HRQoL than those with chronic diseases, and living alone (*t* = 1.53, *p* < 0.05). One-way ANOVA and *LSD* post-hoc tests showed significant differences in HRQoL based on age (*F* = 41.74, *p* < 0.05), with HRQoL decreasing with age among those aged from 60 to 90 but increasing slightly among those over 90. Significant differences were also found based on education level (*F* = 46.40, *p* < 0.05), with older adult individuals with secondary or high school education reporting higher HRQoL than those with other education levels. No significant differences were found based on widowhood (*t* = −0.52, *p* > 0.05).

**Table 1:** Analysis of demographic characteristics on differences in HRQoL (*N* = 729).

Characteristics	*N*	*M*	*SD*	*T/F*	*p*
Gender					
Man	332	70.30	22.42	0.54	>0.05
Woman	397	69.53	16.54		
Age					
60 years ~	308	76.12	18.60	41.74	<0.05
70 years ~	282	69.94	21.12		
80 years ~	126	54.89	2.92		
90 years ~	13	66.35	12.11		
Education					
An illiterate person	315	63.92	11.18	46.40	<0.05
Primary school	187	66.08	24.26		
Junior middle school	145	77.03	20.23		
Technical secondary school and high school	75	91.29	11.51		
College degree or above	7	62.50	14.32		
Widowed					
Yes	202	69.28	20.01	−0.52	>0.05
No	527	70.12	19.21		
Empty nest					
Yes	253	71.39	20.59	1.53	<0.05
No	476	69.08	18.75		
Recreational activities					
Yes	457	69.32	19.92	−1.02	>0.05
No	272	70.82	18.57		
Suffer from chronic diseases					
No	379	81.58	17.08	21.70	<0.05
Yes	350	57.21	12.72		

T, T-test; F,: F-statistic in ANOVA; *p* < 0.05 was considered statistically significant.

### Correlation of life events, social support, and HRQoL

Table 2 shows the correlation between negative life events, social support, and HRQoL among the rural older adult. Negative correlations were found between life events and HRQoL (*r* = −0.57, *p* < 0.05), as well as between social support and health-related quality of life among rural older adult people (*r* = −0.46, *p* < 0.05). It showed a significant positive correlation between social support and HRQoL (*r* = 0.32, *p* < 0.05).

**Table 2:** Correlation analysis of HRQoL, life events, and social support in rural older adults (*N* = 729).

Variable	*M*	*SD*	Life events	Social support	HRQoL
Life events	0.83	0.93	1		
Social support	4.44	0.87	−0.57**	1	
HRQoL	69.88	19.43	−0.46**	0.32**	1

**p < 0.05.

### Regression analysis

Based on the research questions, the samples were grouped by age, educational attainment, and whether they were living alone or not. Regression analysis was conducted for each group separately to analyze the impact of life events and social support on HRQoL.

As shown in Table 3, Regression results of age groups showed that among rural older adult people aged 60–89, social support was positively correlated with HRQoL. Life events are negatively correlated with HRQoL. In the group aged 90 and above, social support had no significant effect on HRQoL, but life events still had a significant negative impact on HRQoL. The regression results of educational attainment groups showed that among rural older adult people with illiteracy, primary and junior high school education levels, social support positively predicted HRQoL, and life events negatively predicted HRQoL. For the secondary vocational school and high school education groups, life events positively predicted HRQoL. Social support positively and significantly predicted HRQoL, and life events negatively and significantly predicted HRQoL. However, due to the small sample size, the representativeness of the results was limited. In empty-nest families, social support positively and significantly predicted HRQoL, while life events negatively and significantly predicted HRQoL. In non-empty nest families, social support negatively predicted HRQoL, and life events also negatively predicted HRQoL.

**Table 3:** Grouped regression analysis on HRQoL (*N* = 729).

Grouping variable	*N*	independent variable social support (β)	independent variable life events (β)
Age			
60 years ~	308	−0.21*	−0.61*
70 years ~	282	0.46*	−0.49*
80 years ~	126	0.23*	−0.85*
90 years ~	13	0.33	−0.63*
Education			
An illiterate person	315	0.20*	−0.54*
Primary school	187	0.45*	−0.26*
Junior middle school	145	0.33*	−0.77*
Technical secondary school and high school	75	0.04	0.65*
College degree or above	7	0.58*	−0.81*
Empty nest			
Yes	253	0.55*	−0.36*
No	476	−0.21*	−0.54*

*p < 0.05.

In order to make the discontinuous variable have the characteristics of continuous variable, demographic characteristics variables such as age, education, empty nest or not and the presence of chronic diseases or not were virtualized. And the health-related quality of life of the rural older adult was taken as the dependent variable. According to reference to the regression test of moderating effect (16), life events, social support and the product of life events and social support were taken as independent variables, and gradually included in the regression equation analysis. In the first step, virtualized demographic characteristics variables in model one were taken as independent variables. In the second step, virtualized demographic characteristics variables in model two, life events and social support were taken as independent variables. At the last step, virtualized demographic characteristics variables, life events, social support and the interaction term that life events were multiplied by social support were taken as independent variables in model three.

As shown in Table 4, in the first step, demographic variables significantly predicted HRQoL. In the second step, life events negatively predicted HRQoL (β = −0.21, *t* = −5.73, *p* < 0.05), while social support positively predicted HRQoL (β = 0.22, *t* = 6.46, *p* < 0.05). In the third step, the interaction term that life events were multiplied by social support negatively predicted HRQoL (β = −0.31, *t* = −8.68, *p* < 0.05). It indicates that social support moderates the relationship between life events and HRQoL. The hypothesis Ha was supported.

**Table 4:** Regression analysis with life events as independent variable and health-related quality of life as dependent variable (*N* = 729).

Variable	Model 1 (β)	Model 2 (β)	Model 3 (β)
Age virtualization 1	−0.04	0.23*	0.22**
Age virtualization 2	−0.24*	−0.08	−0.04
Age virtualization 3	−0.06	0.09	0.16*
Education level virtualization 1	−0.45**	−0.31*	−0.22
Education level virtualization 2	−0.20	0.11	0.16
Education level virtualization 3	0.01	0.18	0.32**
Education level virtualization 4	0.10	0.23**	0.36**
Whether the empty-nest virtualization	0.27**	0.22**	0.23**
Have a chronic disease of virtualization or not	−0.65**	−0.45*	−0.41**
Life events		−0.21**	−0.36**
Social support		0.22**	0.32**
The interaction term (life events were multiplied by social support)			−0.31**
*p* (*F* value)	<0.05 (117.27)	<0.05 (130.21)	<0.05 (138.29)
*R^2^*	0.62	0.68	0.71

*p < 0.05; **p < 0.01.

## Discussion

In this study, research findings indicate that that demographic characteristics such as age, education level, and presence of chronic diseases are significant factors influencing the HRQoL of rural older adults. Age growth brings psychological changes, such as loneliness, increased sense of loss, which will make the older adult feel the passage of life and their own aging, produce anxiety, depression and other negative emotions, affecting mental health and quality of life (17). Chronic diseases remain the leading preventable influential factor on HRQoL at the population level (18). Because chronic diseases are often long-term and difficult to cure, and the older adult need to endure the torture and uncertainty of the disease for a long time, which is easy to produce anxiety, depression, fear and other negative emotions (19).

Group regression analysis in this study revealed the differences in the impacts of life events and social support on HRQoL of rural older adult people. Among rural older adult individuals aged 60 to 89, the more social support they received, the higher their quality of life was. Conversely, the more life events they experienced, the lower their quality of life became. In the secondary vocational school and high school education groups, life events had a different impact on HRQoL compared with other groups. The reason could be that these older adult people generally have better economic conditions and stronger coping abilities. Some life events, such as participating in social activities and learning new skills, exerted a positive influence on their quality of life. Empty nest elders rely more greatly on social support to improve their quality of life, and life events have a significant negative impact on their HRQoL. In contrast, in non-empty-nest families, social support negatively predicted HRQoL, which is contrary to common sense. It may be that intergenerational conflicts and other problems within non-empty-nest families weaken the positive role of social support.

Through this study of life events scale, it is found that 729 rural older adult people in this study all think that the 46 life events are negative life events. These psychological pressures not only affect mental health, but also further affect physical conditions and seriously reduce quality of life. Experiencing stressful life events and being exposed to accumulated stressful life events can lead to depression in the older adult (13). The findings of this study confirm that social support is associated with the HRQoL for older adults (20). Negative life events and social support both play important roles in the HRQoL of rural older adults, with social support acting as a moderating variable. This is consistent with the findings in 2024 (10). The results suggest that when rural older adults receive more social support, the impact of negative life events on their HRQoL is reduced. Social support significantly impacted the physical and mental health of Chinese older adults, and the community environment moderated this relationship (21). It is that individuals with more social support were less affected by stressful events. However, when social support is limited, stressful life events significantly affect their HRQoL.

This study confirms that life events significantly impair the health-related quality of life of rural older adults in China, while social support serves as a critical buffer that directly addresses our research question on how these factors interact. For older adult individuals living in rural areas, who face physical decline, psychological instability, and limited social interactions, social support from family, neighbors, and the government is crucial.

Therefore, it is essential to enhance social support for rural older adults. At the family level, families should not only focus on the physical health of older adult individuals, but also pay attention to their psychological and emotional well-being (22). It is essential to enhance emotional support and guidance by encouraging family members to engage in regular, meaningful emotional exchanges with older adult individuals in rural areas. In addition to monitoring their physical well-being, family members should actively inquire about their psychological states and promptly address any negative emotions that arise. At the community level, cultural infrastructure should be upgraded, and a variety of cultural activities should be organized to fulfill the spiritual and cultural needs of the older adult while fostering social interaction among them. And integrated community programs should establish village-level centers combining health services like chronic disease management with social activities such as group exercises (23). Mutual assistance groups should be established to encourage the older adult to support one another and share experiences, thereby cultivating an atmosphere of mutual aid within the community. At the governmental level, economic subsidies for the older adult should be expanded to ensure their basic living requirements are met and to mitigate the adverse effects of financial strain on their quality of life. Efforts should also focus on strengthening the rural healthcare system through increased investment in medical resources and improved chronic disease management services. Regular free health check-ups, home-based medication delivery, and health counseling should be provided to older adult individuals with chronic conditions. Furthermore, policy guidance and support should be enhanced to incentivize societal participation in rural older adult care services. For example, tax breaks and financial incentives could be offered to enterprises and social organizations engaged in rural older adult care, channeling more resources into this domain and elevating the overall quality of care.

This study revealed the influencing factors of HRQoL among rural older adults to provide evidence for improving their health level. However, this study has several limitations. First, the cross-sectional design precludes causal inferences and limits our ability to establish causal relationships between life events, social support, and HRQoL. Longitudinal studies are needed to track temporal changes and confirm causality. Second, findings from rural China may not fully generalize to other populations. Future research should employ longitudinal designs and incorporate objective measures to mitigate these limitations.

## Conclusion

In summary, the HRQoL of rural older adult individuals is influenced by negative life events and social support, with the latter acting as a moderating variable. Consequently, families should provide enhanced emotional support to the older adult. Communities ought to organize a greater variety of social activities tailored to the needs of the older adult, thereby fostering a warm and inclusive social environment. The government should increase funding for older adult care services and refine the social support system. Additionally, it is essential to establish a psychological counseling framework specifically designed for rural older adult populations to assist them in effectively managing life stress. These measures will provide increased social support to rural older adult individuals, alleviating the stress caused by life events and improving their health-related quality of life, which will contribute to achieving the goal of “Healthy China 2030.”

## Data Availability

The datasets presented in this study can be found in online repositories. The names of the repository/repositories and accession number(s) can be found in the article/Supplementary material.
